# Reclassifying *IDUA* c.250G>A (p.Gly84Ser): Evidence for a Possible Pseudodeficiency Allele

**DOI:** 10.3390/ijns11040100

**Published:** 2025-10-27

**Authors:** Christopher Connolly, Rachel Fisher, Chen Yang, Susan Schelley, Bryce A. Mendelsohn, Chung Lee, Ayesha Ahmad

**Affiliations:** 1 Department of Pediatrics, Division of Pediatric Genetics, University of Michigan, 1500 E Medical Center Drive, Ann Arbor, MI 48109, USA; connolch@med.umich.edu (C.C.); chnyang@med.umich.edu (C.Y.); ayeshaah@med.umich.edu (A.A.); 2 Department of Pathology, Michigan Medicine, 1500 E Medical Center Drive, Ann Arbor, MI 48109, USA; 3 Department of Pediatrics, Division of Medical Genetics, Stanford Medicine Children’s Health, 453 Quarry Road, Palo Alto, CA 94304, USA; 4 Department of Medical Genetics, Kaiser Permanente, Northern California, 3505 Broadway, Oakland, CA 94611, USA

**Keywords:** newborn screening, lysosomal storage disorders, pseudodeficiency alleles, mucopolysaccharidosis, type I, MPS I, Hurler syndrome

## Abstract

Accurate variant classification is crucial for newborn screening (NBS) to prevent missed diagnoses or unnecessary interventions. The *IDUA* gene variant denoted as c.250G>A (p.Gly84Ser) has been identified in individuals with positive NBS for Mucopolysaccharidosis Type I (MPS I). This variant has conflicting pathogenicity reports including one publication classifying this variant as associated with a severe MPS I phenotype; therefore, we aim to clarify the clinical significance of this variant by presenting a case series describing three individuals, each homozygous for c.250G>A (p.Gly84Ser), identified in Michigan and California. All patients in this case series had low alpha-iduronidase (IDUA) enzyme activity with normal or mildly elevated glycosaminoglycans (GAGs) in blood or urine not falling into the range or pattern seen for affected individuals. None of these patients have developed clinical features of MPS I during follow-up ranging up to 3.5 years of age. Review of functional and population data supports a pseudodeficiency effect, resulting in no need for treatment. Based on our experience with three patients all homozygous for c.250G>A (p.Gly84Ser), despite causing low in vitro IDUA activity, homozygosity for the *IDUA* gene variant denoted as c.250G>A (p.Gly84Ser), does not cause symptoms of MPS I and may represent a pseudodeficiency allele. Caution should be exercised in newborns with this variant to help reduce unnecessary interventions and alleviate the psychosocial and economic consequences of false-positive NBS results, particularly for the South Asian population.

## 1. Introduction

Mucopolysaccharidosis Type I (MPS I) is an autosomal recessive lysosomal storage disorder (LSD) caused by deficiency of alpha-iduronidase (IDUA), which is essential for the degradation of glycosaminoglycans (GAGs) such as dermatan sulfate (DS) and heparan sulfate (HS) [1,2,3,4,5,6]. Newborn screening (NBS) for MPS I was added to the Recommended Uniform Screening Panel in 2016 [2,3,6,7]. Enzyme replacement therapy (ERT) combined with hematopoietic stem cell transplantation (HSCT), demonstrates benefits when initiated early in life for severe phenotypes [1,2,3,4,5,6]. However, the introduction of NBS for MPS I has created inevitable learning curves. Pseudodeficiency allele, a genetic variant resulting in decreased in vitro enzyme activity without causing clinical symptoms [3], is common in MPS I NBS, and necessitates second tier testing to help mitigate the risk of false-positive results [1,2,6]. Regular variant and patient re-evaluation, second tier testing, and confirmatory testing, enhances understanding of natural history, incidence, and symptom variability based on genotype.

This article presents a NBS MPS I positive case series involving the variant in the *IDUA* gene, denoted as c.250G>A (p.Gly84Ser), which has conflicting classification in the medical literature. Specifically, this variant has been classified as a variant of unclear significance, with one report raising questions of pathogenicity associated with severe phenotypes [7,8,9]. Careful review of NBS results, second tier results, confirmatory testing, patient evaluation, cascade family and sibling testing, and review with other NBS programs enhanced understanding of this variant. We propose that this variant is most indicative of a pseudodeficiency allele, particularly prevalent in the South Asian population.

## 2. Materials and Methods

**Figure 1:**
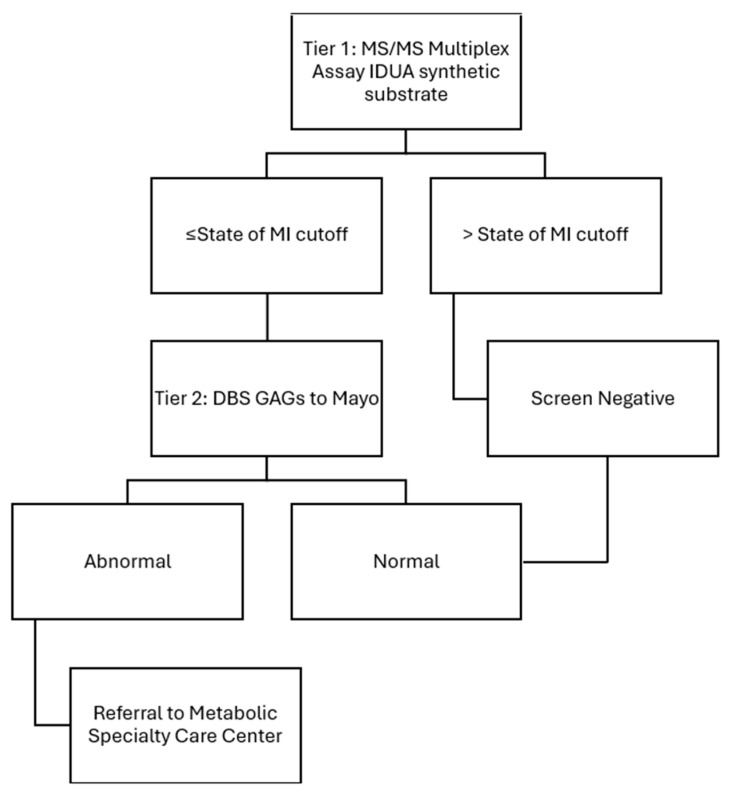
State of Michigan’s MPS I newborn screening protocol courtesy of Michigan State Newborn Screening Program.

**Figure 2:**
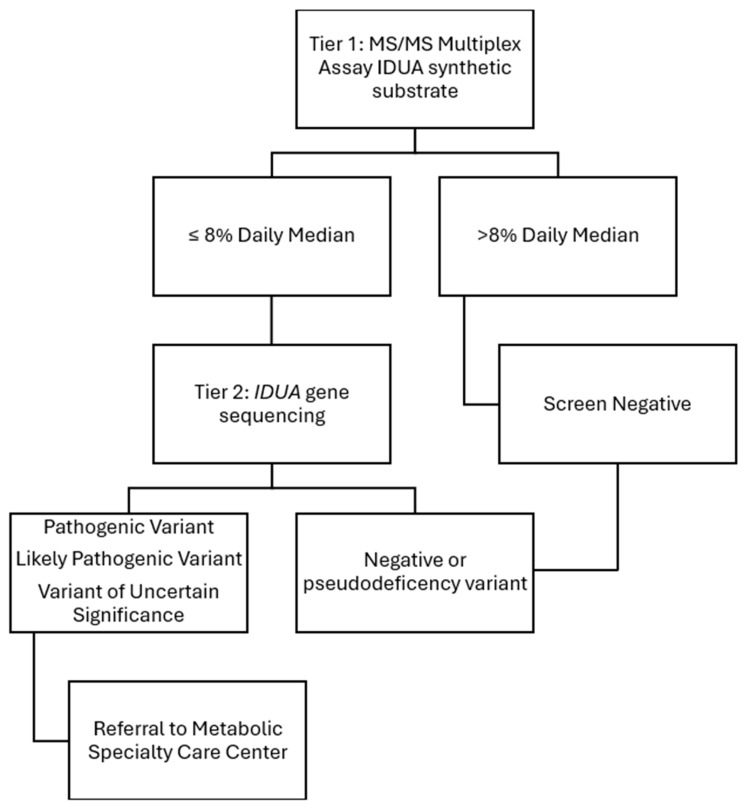
State of California’s MPS I newborn screening protocol courtesy of California State Newborn Screening Program.

The patients in this case series were initially identified via NBS and managed locally in their respective states. Notably, the newborn screening algorithms are different in the two states where these cases took place, shown in Figure 1 and Figure 2 for the state of Michigan and state of California’s newborn screening protocols, respectively.

**Figure 3:**
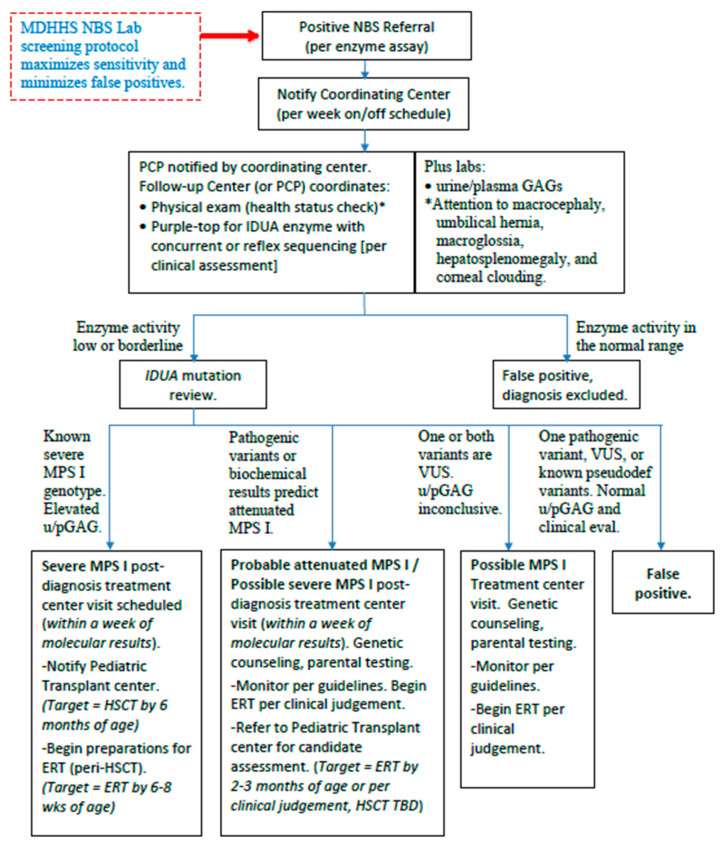
Michigan MPS I diagnosis and management following positive newborn screen (version 2, year 2025). Courtesy of Michigan Department of Health and Human Services. * clinical features noted if a physical exam is being completed.

Michigan’s newborn screening algorithm currently uses mass spectrometry to test IDUA enzyme activity at the state level. If enzyme activity is less than or equal to the determined cut-off, then dried blood spot (DBS) quantitative GAGs levels are completed through Mayo Biochemical Genetics Laboratory (Mayo) as second tier testing. Confirmatory testing is pursued only if second tier testing is positive and includes urine quantitative GAGs and *IDUA* gene analysis. Michigan’s diagnosis and management algorithm following a positive newborn screening result is illustrated in Figure 3.

For case 1 and case 2, urine GAGs were completed at Mayo Biochemical Genetics Laboratory and *IDUA* gene analysis was completed by the Michigan Medical Genetics Laboratories. California’s newborn screening algorithm completes IDUA enzyme activity by mass spectrometry and *IDUA* gene analysis at the state level. Confirmatory testing includes repeat IDUA enzyme analysis and dried blood spot GAGs through the Mayo Biochemical Genetics Laboratory. Case 3 additionally included urine quantitative GAGs and thin layer chromatography through Stanford University as well as Greenwood Genetics Laboratory.

Due to concerns of potential pseudodeficiency of the *IDUA* gene variant denoted as c.250G>A (p.Gly84Ser), the Michigan NBS medical follow-up team contacted the Mayo Biochemical Genetics Laboratory and requested to be connected with clinicians across the country that have submitted samples confirmed to have the variant in question. The cases below demonstrate all the known *IDUA* gene variant denoted as c.250G>A (p.Gly84Ser) patients in the United States that had confirmatory testing sent to Mayo Biochemical Genetics Laboratory. A mass email was sent to the National Society of Genetic Counselors to determine if further cases of this variant were known, but no responses were received from this line of inquiry.

## 3. Results

The following is a compilation of cases of patients identified to be homozygous for the *IDUA* gene variant denoted as c.250G>A (p.Gly84Ser).

### 3.1. Case I

A strong positive result from a newborn screen performed on an infant of South Asian descent of a consanguineous couple was received at a Michigan follow-up center, based on both low alpha-iduronidase (IDUA) enzyme level (0.13 umol/L/h; reference range > 1.75) and abnormal second tier test results (Table 1) with elevated DBS GAGs levels. Second tier testing for this patient demonstrated elevated dermatan sulfate levels of 493 nmol/L (reference range ≤ 200), while heparan sulfate levels were normal.

Physical examination of the patient completed on day thirteen of life and re-evaluated at six weeks of age was normal and revealed no clinical signs consistent with a diagnosis of MPS I. There was no evidence of macrocephaly, macroglossia, proptosis, skeletal concerns, or hepatosplenomegaly.

Confirmatory biochemical testing through Mayo Biochemical Genetics Laboratory revealed low IDUA enzyme activity of 0.36 nmol/h/mg prot (reference range ≥ 2.06). Urine GAGs analysis (Mayo) demonstrated normal dermatan sulfate levels and mildly elevated heparan sulfate levels of 0.72 mg/mmol Cr (reference range ≤ 0.50) (Table 1).

This patient’s DNA was sequenced on the Illumina TruSight One Expanded Exome platform (6794 genes; mean coverage 364.4×). Full *IDUA* gene analysis reported the patient to be homozygous for a variant of uncertain clinical significance denoted as c.250G>A (p.Gly84Ser). A subsequent exome-wide tertiary analysis using the Emedgene model (version 38.0.2) did not reveal any potential genetic modifiers for the MPS I phenotype, using four specific HPO filtering terms (diminished tissue alpha-L-iduronidase activity, dermatan sulfate excretion in urine, heparan sulfate excretion in urine, and abnormal circulating heparan sulfate level). Targeted Sanger analysis was completed on the patient’s mother and father and confirmed their heterozygous status for the variant. IDUA enzyme analysis completed on both parents was normal with activity above the affected range.

### 3.2. Case 2

The index patient’s older brother was evaluated as part of sibling testing. From his initial NBS result, IDUA enzyme activity was low (<2.77 umol/L/h; reference range > 4.89, using digital microfluidics at the time in the MI NBS state lab), leading to second tier testing per the Michigan NBS algorithm. Second tier testing was considered negative; therefore, his newborn screen was reported as normal. However, upon review of his second tier NBS GAGs, dermatan sulfate level was mildly elevated, 493 nmol/L (reference range ≤ 200), while heparan sulfate levels were normal. This patient underwent an evaluation including physical exam and medical history collection at the age of two years eight months old. There were no concerns for developmental delay and physical examination was normal, with no concerns of hypertrichosis, corneal clouding, thickening of facial features (i.e., nasal alae), organomegaly, or other characteristic features of MPS I. IDUA enzyme activity (Mayo) collected at that visit was low, 0.09 nmol/h/mg Prot (reference range ≥ 2.06), and blood GAGs (Mayo) were normal (Table 1). Molecular testing revealed that he was also homozygous for c.250G>A (p.Gly84Ser). Targeted Sanger analysis was completed on the patient’s mother and father and confirmed their heterozygous status for the variant. IDUA enzyme analysis completed on both parents was normal with activity above the affected range.

### 3.3. Case 3

A full-term infant of South Asian descent had a positive newborn screen for MPS I in the state of California. IDUA enzyme analysis was low (0.517 umol/L/h; reference range ≥ 1.018). The result was less than the cut-off of the daily median per the California state newborn screening algorithm for MPS I; therefore, a referral was made for follow-up testing. Sequence analysis of the *IDUA* gene showed homozygosity for c.250G>A (p.Gly84Ser). Familial site-specific molecular testing indicated that both parents were heterozygous of this variant. Confirmatory testing of the proband included IDUA enzyme activity in leukocytes (Mayo) 0.22 nmol/h/mg Prot (≥2.06). Whole blood GAGs analysis (Mayo) showed normal concentrations of dermatan, heparan, and keratan sulfates. Urine GAGs quantitative analysis (Stanford) was normal at 34.3 mg/mmol cr (<48.0mg/mmol cr) with no abnormal bands on thin layer chromatography. Follow-up testing at seven and thirteen months of age showed normal blood and urine GAGs analysis. At the last follow-up visit at three-and-a-half years of age, the patient’s growth and development were normal, without any clinical features of MPS I. Urine GAGs analysis (Greenwood Genetics) was normal except for slightly elevated heparan sulfate of 2.29 g/mol crnn (0.00–2.24) reported by the lab as not clinically significant (Table 1).

## 4. Discussion

In individuals affected by severe MPS I, the incomplete breakdown of GAGs leads to their accumulation in cells, tissues, and bodily fluids, resulting in a variety of symptoms including coarse facial features (thickening of the calvarium, alae nasi, lips, ear lobules), hepatosplenomegaly, hernias, corneal clouding, hypertrichosis, arthropathy, and developmental delays, presenting within the first year of life [1,2,3,4,5,6].

**Figure 4:**
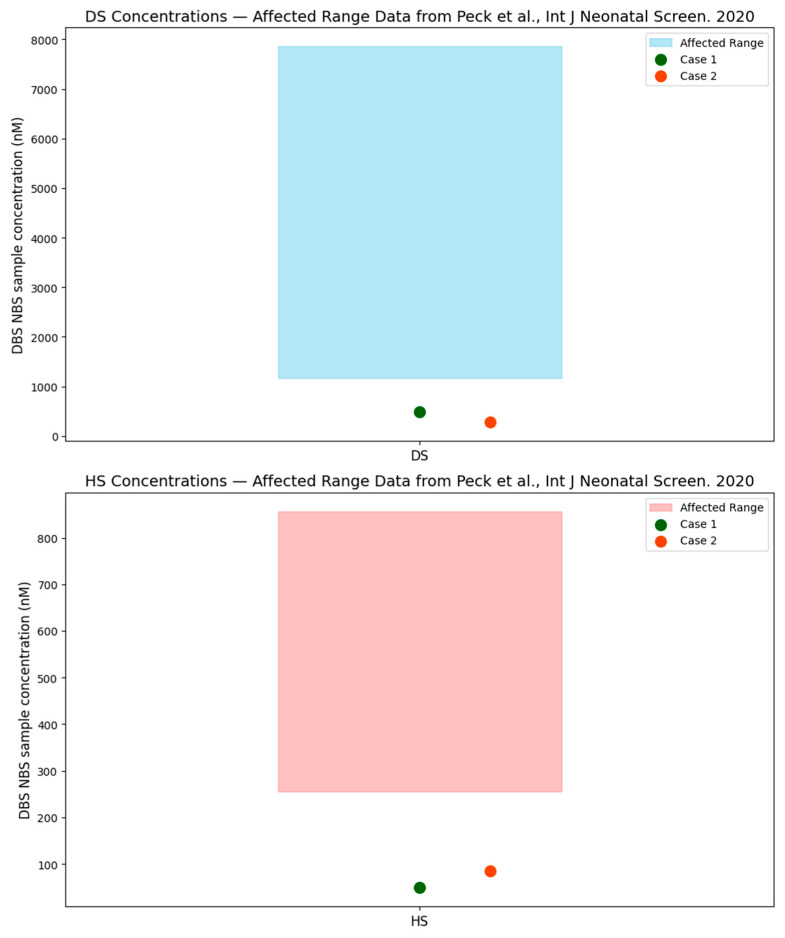
Case 1 and Case 2 DBS, NBS, DS, and HS levels compared to the typically affected levels presented in the Peck, D.S.; Lacey, J.M.; White, A.L.; Pino, G.; Studinski, A.L.; Fisher, R.; Ahmad, A.; Spencer, L.; Viall, S.; Shallow, N.; et al. Incorporation of Second-Tier Biomarker Testing Improves the Specificity of Newborn Screening for Mucopolysaccharidosis Type I. *Int. J. Neonatal Screen.* **2020**, *6*, 10. https://doi.org/10.3390/ijns6010010 [6].

The recent literature has underscored the importance of incorporating second tier tests for newborn screening of MPS I [1,2], specifically targeting the levels of DS and HS in dried blood spots [2,6]. Peck et al., published the use of second tier biomarkers (DS and HS) to improve the specificity of newborn screening for MPS I. In the Peck et al. study, all samples from infants with two pathogenic/likely pathogenic variants in trans had very high concentrations of DS (range: 1167–7859 nM; median 2795 nM, reference range; newborn ≤ 2 weeks: ≤200 nM; >2 weeks: ≤130 nM.) and HS (range 255–857 nM; median 432 nM, reference range; newborn ≤ 2 weeks: ≤96 nM; >2 weeks: ≤95 nM.). Conversely, patients with pseudodeficiency or carriers of pathogenic variants typically had markedly lower levels of these biomarkers. Mild GAGs elevations are frequently seen in NBS samples and are not associated with MPS I [7]. Of note, reference ranges for DBS GAGs vary by lab and within the same lab change over time as additional NBS data is collected. Figure 4 plots the DBS, NBS, DS, and HS levels in the two cases presented in this case series (for which DBS, DS, and HS were available) compared to the typically affected levels presented in the Peck et al. paper, which demonstrates that these cases are well outside of expected affected range. In attenuated cases of MPS I, which have been identified on the newborn screen, GAGs fall within the abnormal range and do not normalize over time.

When IDUA enzyme activity is solely considered in diagnostic evaluation, the presence of pseudodeficiency alleles leads to low predictive value and high false-positive referrals for MPS I NBS [1,2,6]. Pseudodeficiency in MPS I is also more prevalent in people of African descent, leading to increased economic and psychosocial burdens from false-positive screening results in minorities [3,6]. The adoption of second tier testing by newborn screening programs has become crucial in minimizing false-positive results associated with pseudodeficiency alleles and downstream health disparities [1,2,6,10].

The specific variant identified in this case series, c.250G>A (p.Gly84Ser), involves the substitution of a glycine with a serine at the 84th amino acid of the IDUA protein and is classified as of uncertain clinical significance (ClinVar ID: 726495). The highest population frequency of this variant is 0.3% in South Asians, with three homozygotes reported in gnomAD v4.1 [11] implying a possible benign phenotype. In silico predictors, including REVEL and BayesDel_noAF, report scores consistent with a moderate pathogenic variant. Therefore, when ClinGen Lysosomal Diseases Expert Panel Specifications to ACMG/AMP Variant Interpretation Guidelines for *IDUA* [12] are applied, only BS1 (Allele frequency is greater than expected for disorder) and PP3_Moderate (multiple lines of computational evidence support a deleterious effect on the gene or gene product) criteria are met. In accordance with ACMG variant interpretation guidelines for *IDUA*, this variant is best described as a variant of uncertain significance (VUS).

One previous publication [6] describes an individual homozygous for this variant who underwent a hematopoietic stem cell transplant at an early age. This patient was reported to have coarse facial features. No other specific clinical manifestations of MPS I were reported in this publication, and no additional follow-up information is available for this patient. No other publications upon review have reported phenotype information; however, this variant has been reported to be associated with low IDUA enzyme activity [8,9].

A recent functional assessment of *IDUA* variants of uncertain significance provides additional support that c.250G>A (p.Gly84Ser) is a pseudodeficiency allele [12]. The relative specific activity of c.250G>A (p.Gly84Ser) was 16% higher than known variants associated with attenuated MPS I such as p.Pro533Arg and was even higher than p.Ala79Thr, a known pseudodeficiency allele. To the extent that cell model activity predicts activity in vivo, this missense variant clearly clusters with other VUS and pseudodeficiency variants rather than pathogenic variants [12]. In uncertain cases, serial measurements of DS and HS over time could provide valuable insights into the dynamics of these biomarkers on DBS and their potential clinical implications. In the variant presented in this case series, GAGs were normal or normalized after time, not consistent with a diagnosis of MPS I. Caution should be exercised before proceeding to stem cell transplantation in newborns without clinical features clearly suggesting a severe form of MPS I.

## 5. Conclusions

The cases presented here describe three individuals who are homozygous for c.250G>A (p.Gly84Ser), with low IDUA enzyme activity, DS, and HS levels that fall within the unaffected range and no clinical symptoms. The ages of these patients range from newborn to three-and-a-half years old; therefore, if severely affected, symptoms would be expected. In conclusion, the variant c.250G>A (p.Gly84Ser), common in the South Asian population, may represent a pseudodeficiency allele. Cautious follow-up is recommended before proceeding with invasive interventions and to avoid confusion in diagnosing patients with MPS I and unnecessary medical therapies such as ERT and HSCT.

## Figures and Tables

**Table 1:** Summary of testing in cases.

	NBS Biochemical Testing					Confirmatory Biochemical Testing					
Case #	State of NBS	Age of Patient at Time of NBS	Alpha-Iduronidase (IDUA) Enzyme Level	Dermatan Sulfate Level (Dried Blood Spot)	Heparan Sulfate Level (Dried Blood Spot)	Age of Patient at Time of Confirmatory Testing	Alpha-Iduronidase (IDUA) Enzyme Level	Dermatan Sulfate Level (Urine)	Heparan Sulfate Level (Urine)	Dermatan Sulfate Level (Blood)	Heparan Sulfate Level (Blood)
1	MI	25 hours	**0.13** umol/L/h (ref >1.75 umol/L/h)	**493** nmol/L (ref ≤200 nmol/L)	42 nmol/L (ref ≤96 nmol/L)	12 days	**0.36** nmol/h/mg Prot (ref ≥2.06 nmol/h/mg)	0.44 mg/mmol Cr (Urine; ref ≤1.00 mg/mmol Cr)	**0.72** mg/mmol Cr (Urine; ref ≤0.50 mg/mmol Cr)	N/A	N/A
			N/A	N/A	N/A	6 weeks		0.75 mg/mmol Cr (Urine; ref ≤1.00 mg/mmol Cr)	**1.19** mg/mmol Cr (Urine; ref ≤0.50 mg/mmol Cr)	N/A	N/A
2	MI	25 hours	**<2.77** umol/L/h (ref >4.89 umol/L/h)	**285** nmol/L (ref ≤200 nmol/L)	85 nmol/L (ref ≤96 nmol/L)	2.75 years	**0.09** nmol/h/mg Prot (ref ≥2.06 nmol/h/mg)	N/A	N/A	38 nmol/L (Blood spot; ref ≤130 nmol/)	68 nmol/L (Blood spot; ref ≤95 nmol/)
3	CA	43 hours	**0.517** umol/L/h (ref ≥1.018)	N/A	N/A	3 weeks	**0.22** nmol/h/mg Prot (ref ≥2.06 nmol/h/mg)	Normal total GAGs and fractionation by TLC	Normal total GAGs and fractionation by TLC	65 nmol/L (Whole blood; ref ≤130 nmol/)	32 nmol/L (Whole blood; ref ≤95 nmol/)

## Data Availability

The original contributions presented in this study are included in the article. Further inquiries can be directed at the corresponding author(s).

## Abbreviations

The following abbreviations are used in this manuscript:

NBS	Newborn Screening
IDUA	Alpha-Iduronidase
MPS I	Mucopolysaccharidosis Type I
GAGs	Glycosaminoglycans
ERT	Enzyme Replacement Therapy
DBS	Dried Blood Spot
HSCT	Hematopoietic Stem Cell Transplantation
DS	Dermatan Sulfate
HS	Heparan Sulfate
